# Automated urine sediment analyzers underestimate the severity of hematuria in glomerular diseases

**DOI:** 10.1038/s41598-021-00457-6

**Published:** 2021-10-25

**Authors:** Won Seok Yang

**Affiliations:** Division of Nephrology, Department of Internal Medicine, Asan Medical Center, University of Ulsan College of Medicine, 88 Olympic-ro 43-gil, Songpa-gu, Seoul, 05505 Republic of Korea

**Keywords:** Medical research, Nephrology

## Abstract

Hematuria, either glomerular or extraglomerular, is defined as 3 or more red blood cells (RBCs)/high power field. Currently, urinalyses are commonly performed using automated urine sediment analyzers. To assess whether RBC counting by automated urine sediment analyzers is reliable for defining hematuria in glomerular disease, random specimen urinalyses of men with nephritic glomerular disease (7674 urinalyses) and bladder cancer (12,510 urinalyses) were retrospectively reviewed. Urine RBCs were counted by an automated urine sediment analyzer based on flow cytometry (UF-1000i, Sysmex Corporation) or digital image analysis (Cobas 6500, Roche Diagnostics GmbH). In about 20% of urine specimens, the specific gravity was less than 1.010, making the RBC counts unreliable. In the urine specimens with specific gravity ≥ 1.010, RBC counts measured using either UF-1000i or Cobas 6500 were well correlated with the positive grades in the dipstick blood test. However, at a trace, 1+, or higher positive dipstick tests for blood, RBC counts were graded significantly lower in glomerular disease than in bladder cancer. The findings suggest that RBC counting by UF-1000i or Cobas 6500 underestimates the severity of hematuria in glomerular disease, possibly because dysmorphic RBCs in glomerular disease are susceptible to hemolysis and/or fail to be properly recognized.

## Introduction

Hematuria can occur in glomerular diseases as well as extraglomerular diseases, including urologic malignancies. It is detected in the urinalysis by either a dipstick test or microscopic examination of urine sediment. The urinary dipstick test detects the peroxidase-like activity of heme in red blood cells (RBCs) and is very sensitive for hematuria, but may not be specific^1,2^. Thus, hematuria is defined by the number of RBCs in the urine sediment, i.e., 3 or more RBCs/high power field (HPF)^3,4^.

In the past, RBCs in the urine were counted by manual microscopic examination, but it is time-consuming and labor-intensive. For rapid and efficient urinalysis, automated urine sediment analyzers were introduced more than 20 years ago and have taken over this task in clinical laboratories^5,6^.

The current automated urine sediment analyzers identify RBCs by flow cytometry (for example, UF-1000i urine analyzer, Sysmex Corporation, Kobe, Japan) or analysis of digitalized images of the urine sediment (for example, Cobas 6500 urine analyzer, Roche Diagnostics GmbH, Mannheim, Germany)^7,8^. The analyzers have good performance for the identification of isomorphic RBCs as in extraglomerular hematuria^9^.

In glomerular disease, the presence of hematuria is a key factor in differentiating nephritic disease from nephrotic disease. Recently, more attention has been paid to hematuria in glomerular disease because hematuria was shown to have a negative prognostic impact^10^.

Glomerular hematuria is different from extraglomerular hematuria in terms of the RBC morphology. RBCs from extraglomerular hematuria have an isomorphic appearance as seen in peripheral blood smears, whereas RBCs of glomerular origin are small and have a dysmorphic appearance with marked variability in size and shape^11^. The morphologic alterations of RBCs occur due to mechanical trauma taking place while they pass through the glomerular basement membrane or the osmotic stress due to high or low osmolarity within the renal tubules^12^.

Glomerular hematuria is also defined as the presence of ≥ 3 or 5 RBCs/HPF^13–21^. To assess whether RBC counting by automated urine sediment analyzers is reliable for defining hematuria in glomerular disease, this study compared urine RBC counts of patients with nephritic glomerular disease and those with bladder cancer at each positive grade in a dipstick blood test in the urinalyses performed using two automated urine sediment analyzers (UF-1000i urine analyzer and Cobas 6500 urine analyzer).

## Methods

### Patients

This study included adult patients (≥ 18 years) who were diagnosed with nephritic glomerular disease via a kidney biopsy and diagnosed with bladder cancer via a bladder biopsy or transurethral resection of bladder tumor at Asan Medical Center (Seoul, Korea), a tertiary hospital; the patients’ medical records were retrospectively reviewed. Between January 2011 and November 10, 2016, urine sediment examinations were carried out using the UF-1000i urine analyzer (Sysmex Corporation). Thereafter, the instrument for urinalysis was changed to the Cobas 6500 urine analyzer (Roche Diagnostics GmbH). For the analysis of the UF-1000i urine analyzer data, the urinalyses of the patients with glomerular disease performed between January 2011 and October 2016 and the urinalyses of the patients with bladder cancer performed between January 2013 and December 2015 were collected. For the analysis of the Cobas 6500 urine analyzer data, the urinalyses of the patients with glomerular disease performed between December 2016 and December 2020 and the urinalyses of the patients with bladder cancer performed between January 2017 and December 2019 were collected.

For the patients with glomerular disease, some urinalyses were conducted the day after the kidney biopsy, which may have caused extraglomerular hematuria. Thus, only urinalyses from outpatients, and not inpatients, were included. In young women with glomerular disease, extraglomerular hematuria derived from menstrual blood may contaminate the glomerular hematuria; thus, only men were included. For the same reason, patients with both bladder cancer and glomerular disease were also excluded. In the case of patients with bladder cancer who had undergone radical cystectomy with formation of an ileal conduit or neobladder during the study period, only those urinalysis data obtained prior to the cystectomy were included. Finally, the urinalyses of gross hematuria, identified via the red, reddish, or orange hue of the urine, were excluded.

This study was approved by the Institutional Review Board (IRB) of Asan Medical Center (IRB No. S2021-0242-0001); the requirement of obtaining informed consent was waived owing to the retrospective study design. All methods were performed in accordance with the Declaration of Helsinki.

### Laboratory parameters

Urine specimens were randomly obtained at the outpatient clinic and transported to the clinical laboratory without preservatives. The laboratory aimed to report the urinalysis results within 1 h of sampling.

Urinary RBCs counted via either the UF-1000i urine analyzer or the Cobas 6500 urine analyzer were reported as follows: grade 1 (0–2 RBCs/HPF), grade 2 (3–5 RBCs/HPF), grade 3 (6–10 RBCs/HPF), grade 4 (11–20 RBCs/HPF), grade 5 (21–30 RBCs/HPF), grade 6 (31–100 RBCs/HPF) and grade 7 (> 100 RBCs/HPF).

The presence of blood and the pH of the urine were measured via a dipstick test. The urine color and the dipstick test results were read via an automatic reader. The Urisys 2400 automated urine test strip analyzer (Roche Diagnostics GmbH) was used to read the dipstick tests of the urine specimens examined using the UF-1000i urine analyzer, whereas the Cobas 6500 urine analyzer was used to conduct the urine dipstick test as well as the urine RBC counting using two modules, the Cobas u 601 for the urine dipstick and the Cobas u 701 for urine microscopy. Test strips in the Urisys 2400 cassettes and the cobas u packs were used with the Urisys 2400 analyzer and the Cobas u 601 analyzer, respectively. In both analyzers, dipstick hematuria was graded as −, ±, 1+, 2+, 3+, and 4+. The pH of the urine was categorized as follows; 5, 6, 6.5, 7, 8 and 9.

The Urisys 2400 and Cobas u 601 urine analyzers both contain a built-in refractometer, and the specific gravity (SG) was measured via refractometry. The scale of urine SG ranged from 1.000 to 1.050. In dilute urine, the RBCs absorb water, swell, and may rupture. In concentrated urine, the RBCs tend to shrink and become crenated. A urine SG of 1.010 corresponds to approximately 300 mOsm/kg, similar to the osmolarity of plasma^22^, and a urine SG > 1.020 often indicates dehydration^23^. In this study, therefore, urine samples with SG < 1.010 and > 1.020 were considered dilute and concentrated urine, respectively.

### Statistical analysis

The data are expressed as the median (interquartile range). The values obtained for the two groups were compared using the Mann*–*Whitney U test. To measure the strength of association between the positive degree on the dipstick blood test and the urine RBC count, Spearman’s rank-order correlation was used. The prevalence of categorical variables was compared between the two groups using the chi-squared test or Fisher’s exact test. Statistical analyses were performed using SPSS version 21 (IBM Co., Armonk, NY, USA). *p* values less than 0.05 were considered statistically significant.

## Results

### Patient characteristics

In the data obtained via the UF-1000i urine analyzer, 330 patients were diagnosed with nephritic glomerular disease (235, IgA nephropathy; 39, pauci-immune crescentic glomerulonephritis; 35, lupus nephritis; 13, membranoproliferative glomerulonephritis; 6, Henoch-Schönlein purpura; and 2, postinfectious glomerulonephritis). The median age at diagnosis was 47 (28–58) years. The control group consisted of 967 patients who were diagnosed with bladder cancer (958, transitional cell carcinoma; 4, bladder invasion of prostate carcinoma; 3, urachal carcinoma; 1, spindle cell neoplasm; and 1, metastatic sigmoid colon carcinoma). The median age at diagnosis was 67 (59–74) years (p < 0.001 compared with glomerular disease). The number of urinalyses was 9 (3–17) among the patients with glomerular disease and 5 (3–7) among the patients with bladder cancer.

In the data obtained via the Cobas 6500 urine analyzer, 385 patients were diagnosed with glomerular disease (266, IgA nephropathy; 57, pauci-immune crescentic glomerulonephritis; 36, lupus nephritis; 13, Henoch-Schönlein purpura; 11, membranoproliferative glomerulonephritis; and 2, postinfectious glomerulonephritis). The median age at diagnosis was 46 (30–58) years. The control group consisted of 1087 patients who were diagnosed with bladder cancer (1064, transitional cell carcinoma; 7, urachal carcinoma; 7, neuroendocrine small cell carcinoma; 6, bladder invasion of prostate carcinoma; 1, mucinous adenocarcinoma; 1, pleomorphic undifferentiated sarcoma; and 1, invasive poorly differentiated carcinoma). The median age at diagnosis was 69 (60–75) years (p < 0.001 compared with glomerular disease). The number of urinalyses was 9 (4–13) among the patients with glomerular disease and 6 (4–9) among the patients with bladder cancer.

### Distributions of urine SG and pH

**Figure 1:**
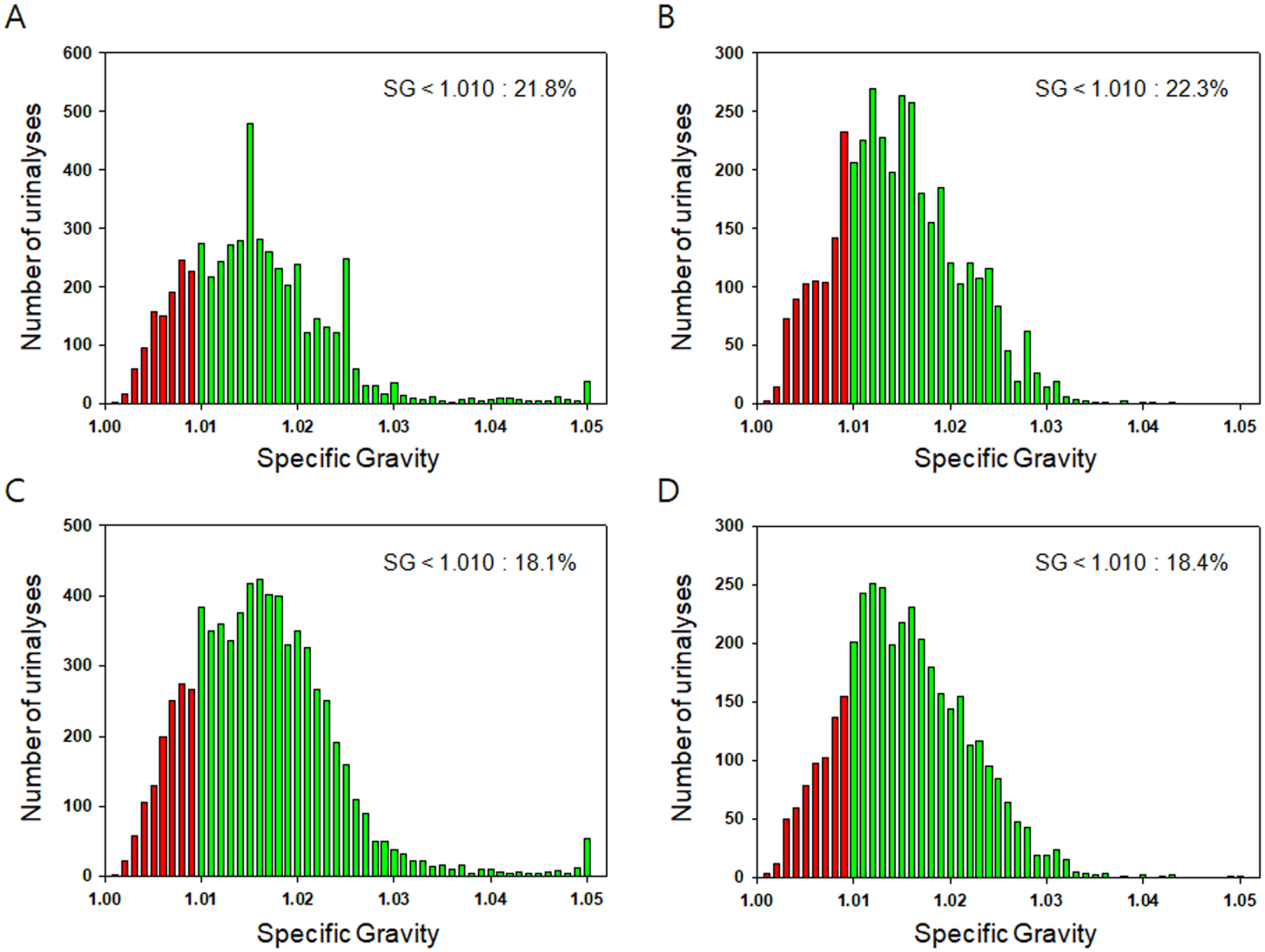
Specific gravities (SG) of urine specimens measured using the UF-1000i urine analyzer ((**A**) bladder cancer; (**B**) glomerular disease) and the Cobas 6500 urine analyzer ((**C**) bladder cancer; (**D**) glomerular disease).

The SG of urine affects the morphology of RBCs and may thus alter the identification of RBCs via automated urine sediment analyzers. The distribution of the urine SG in each group is shown in Fig. 1.

In the data obtained via the UF-1000i urine analyzer, the SG of urine from patients with bladder cancer and glomerular disease was 1.015 (1.010–1.020) and 1.014 (1.010–1.019) (p < 0.001), respectively. The frequency of a dilute urine specimen with SG < 1.010 was 21.8% (1148/5271) among patients with bladder cancer and 22.3% (866/3892) among patients with glomerular disease. In contrast, the frequency of a concentrated urine specimen with SG > 1.020 was 21.7% (1142/5271) among patients with bladder cancer and 18.9% (737/3892) among patients with glomerular disease (p < 0.001).

In the data obtained via the Cobas 6500 urine analyzer, the SG of urine from patients with bladder cancer and glomerular disease was 1.016 (1.011–1.020) and 1.015 (1.011–1.020) (p < 0.001), respectively. The frequency of a dilute urine specimen with SG < 1.010 was 18.1% (1310/7239) among patients with bladder cancer and 18.4% (696/3782) among patients with glomerular disease. In contrast, the frequency of a concentrated urine specimen with SG > 1.020 was 24.9% (1806/7239) among patients with bladder cancer and 21.6% (816/3782) among patients with glomerular disease (p < 0.001).

To examine the effects of high and low urine SG values on RBC counting via automated urine sediment analyzers, the RBC counts at each positive grade in the dipstick blood test were compared according to the SG. In most cases, the RBC counts were graded significantly lower in urine specimens with SG < 1.010 than in those with SG 1.010–1.020. High urine SG (> 1.020) did not seem to alter the RBC counting via the UF-1000i urine analyzer, but tended to decrease the RBC counts in patients with glomerular disease when the urinalyses were performed using the Cobas 6500 urine analyzer (Supplementary Tables S1–S4, Supplementary Figs. S1–S4).

Urine pH is another factor that affects the morphology of RBCs. In highly alkaline urine (pH > 9), RBCs may undergo lysis due to swelling of the outer membrane layers^24^. In the data obtained via the UF-1000i urine analyzer, the pH of urine from bladder cancer and glomerular disease was 6 (5–6.5) and 5 (5–6.5) (p < 0.001), respectively. There were 7/5271 (0.13%) urine specimens with pH ≥ 9 among the patients with bladder cancer and 2/3892 (0.05%) among the patients with glomerular disease. In the data obtained via the Cobas 6500 urine analyzer, the pH of urine from bladder cancer and glomerular disease was 6 (5–7) and 5 (5–6) (p < 0.001), respectively. There were 16/7239 (0.22%) urine specimens with pH ≥ 9 among the patients with bladder cancer and 6/3782 (0.16%) among the patients with glomerular disease.

### Correlation between the urinary dipstick blood test and urinary RBC counts

The relation between positive grades in the dipstick test and urinary RBC counts was assessed in urine samples with SG ≥ 1.010 and pH < 9.0.

In the data obtained via the UF-1000i urine analyzer, the Spearman’s correlation coefficient for the correlation between the positive grades in the dipstick test and urine RBC counts was 0.900 (n = 4117, p < 0.001) in bladder cancer and 0.829 (n = 3024, p < 0.001) in glomerular disease, respectively.

In the data obtained via the Cobas 6500 urine analyzer, the Spearman’s correlation coefficient for the correlation between the positive grades in the dipstick test and urine RBC counts was 0.911 (n = 5914, p < 0.001) in bladder cancer and 0.834 (n = 3081, p < 0.001) in glomerular disease, respectively.

### RBC counts at each positive degree of the dipstick blood test

The RBC counts at each positive grade of the dipstick blood test were compared between glomerular disease and bladder cancer in urine samples with SG ≥ 1.010 and pH < 9.0.

In both bladder cancer and glomerular disease, the RBC counts were widely distributed at each degree of positivity in the dipstick test, suggesting that some RBCs undergo lysis before urinalysis or are not recognized (Tables 1, 2).

**Table 1:** The distributions of urine RBC counts measured using the UF-1000i urine analyzer at each positive degree of dipstick blood test in patients with bladder cancer (A) and glomerular disease (B).

Grade (RBCs/HPF)	Dipstick blood test					
	−	±	1+	2+	3+	4+
**A. Bladder cancer**						
7 (> 100/HPF)				1	5	536
6 (31–100/HPF)	1			6	72	343
5 (21–30/HPF)			2	33	77	57
4 (11–20/HPF)			11	108	67	65
3 (6–10/HPF)	3	14	94	105	51	32
2 (3–5/HPF)	19	65	120	73	22	22
1 (0–2/HPF)	1621	194	207	70	12	9
**B. Glomerular disease**						
7 (> 100/HPF)					1	81
6 (31–100/HPF)				1	16	220
5 (21–30/HPF)				8	28	107
4 (11–20/HPF)			9	61	109	201
3 (6–10/HPF)		3	31	87	106	98
2 (3–5/HPF)	14	17	74	117	91	50
1 (0–2/HPF)	761	188	335	148	46	16

*RBCs* red blood cells, *HPF* high power field.

**Table 2:** The distributions of urine RBC counts measured using the Cobas 6500 urine analyzer at each positive degree of dipstick blood test in patients with bladder cancer (A) and glomerular disease (B).

Grade (RBCs/HPF)	Dipstick blood test					
	−	±	1+	2+	3+	4+
**A. Bladder cancer**						
7 (> 100/HPF)		1			17	977
6 (31–100/HPF)	1	1		26	233	422
5 (21–30/HPF)		1	9	59	90	61
4 (11–20/HPF)	2	29	40	119	81	57
3 (6–10/HPF)	23	144	40	76	44	21
2 (3–5/HPF)	217	127	9	66	21	11
1 (0–2/HPF)	2546	249	14	55	18	7
**B. Glomerular disease**						
7 (> 100/HPF)						146
6 (31–100/HPF)					22	303
5 (21–30/HPF)				3	29	130
4 (11–20/HPF)			2	26	56	173
3 (6–10/HPF)		13	2	48	88	88
2 (3–5/HPF)	20	68	13	76	78	81
1 (0–2/HPF)	1006	293	29	158	104	26

*RBCs* red blood cells, *HPF* high power field.

**Figure 2:**
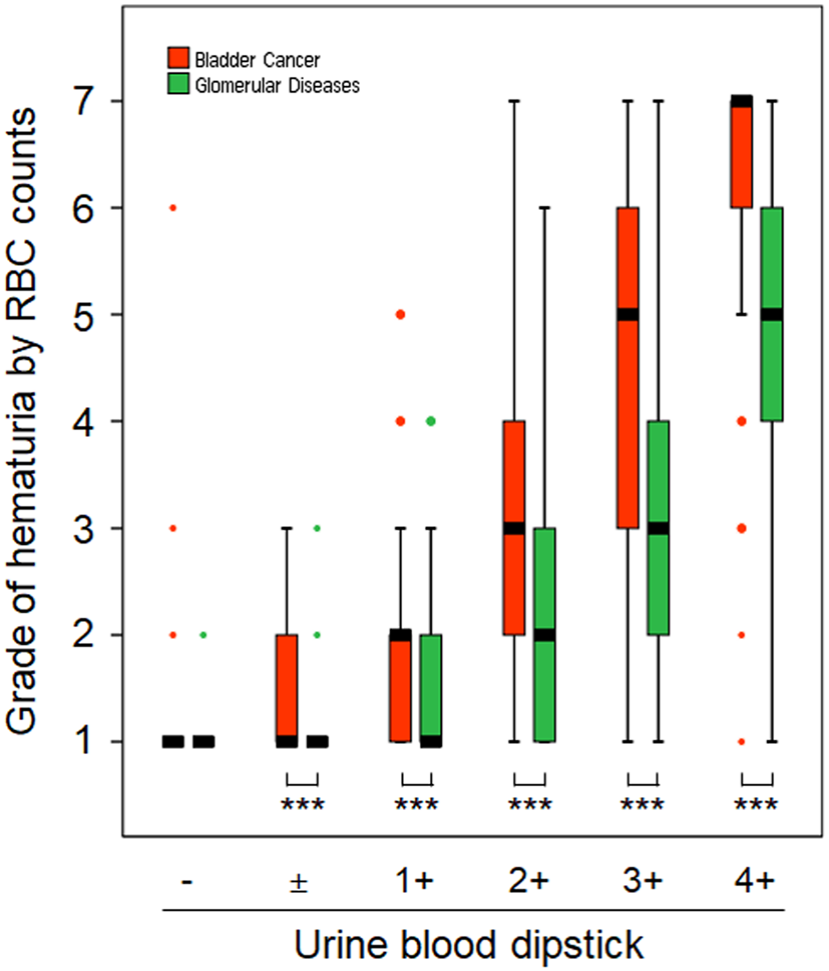
Comparison of hematuria grades measured using the UF-1000i urine analyzer between bladder cancer and glomerular disease at each positive dipstick grade. Vertical boxes, error bars, and dots represent the median with interquartile range, minimum and maximum values, and outliers, respectively (***p < 0.001).

However, at a trace, 1+, or higher positive dipstick tests for blood, RBC counts were graded significantly lower in glomerular disease than in bladder cancer. In the data obtained via the UF-1000i urine analyzer, the grades of RBC counts/positivity in the dipstick test were 1 (1–1)/−, 1 (1–2)/±, 2 (1–2)/1+, 3 (2–4)/2+, 5 (3–6)/3+, and 7 (6–7)/4+ in bladder cancer, and 1 (1–1)/−, 1 (1–1)/±, 1 (1–2)/1+, 2 (1–3)/2+, 3 (2–4)/3+, and 5 (4–6)/4+ in glomerular disease, with the magnitude of difference bigger as the positive grade increased (Fig. 2).

**Figure 3:**
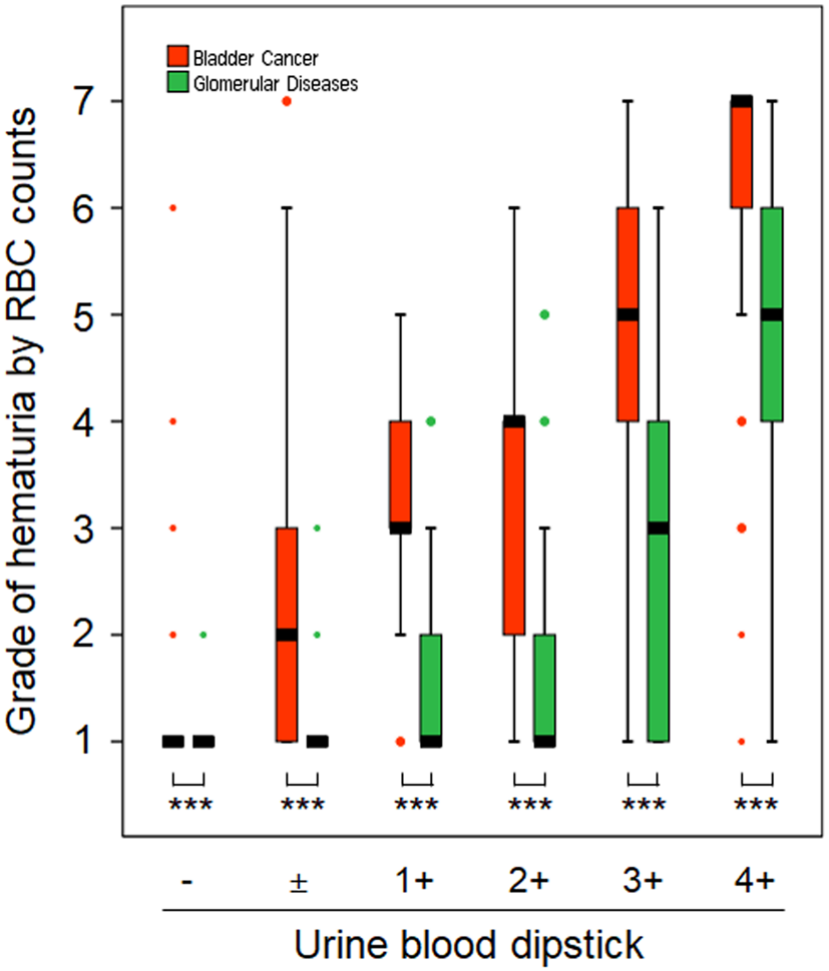
Comparison of hematuria grades measured using the Cobas 6500 urine analyzer between bladder cancer and glomerular disease at each positive dipstick grade. Vertical boxes, error bars, and dots represent the median with interquartile range, minimum and maximum values, and outliers, respectively (***p < 0.001).

The difference was greater in the data obtained via the Cobas 6500 urine analyzer at 1+ or higher positive dipstick tests for blood; 1 (1–1)/−, 2 (1–3)/±, 3 (3–4)/1+, 4 (2–4)/2+, 5 (4–6)/3+, and 7 (6–7)/4+ in bladder cancer, and 1 (1–1)/−, 1 (1–1)/±, 1 (1–2)/1+, 1 (1–2)/2+, 3 (1–4)/3+, and 5 (4–6)/4+ in glomerular disease (Fig. 3).

A dipstick positive result, but a negative microscopic result (RBC 0–2/HPF) was also more frequent in glomerular disease than in bladder cancer: 13.5% in bladder cancer and 26.7% in glomerular disease in the data obtained via the UF-1000i urine analyzer (p < 0.001), and 3.7% in bladder cancer and 18.9% in glomerular disease in the data obtained via the Cobas 6500 urine analyzer (p < 0.001).

Among the urine samples with a negative result in the dipstick test, the frequency of 3 or more RBCs/HPF was similar between bladder cancer (1.4%) and glomerular disease (1.8%) in the data obtained via the UF-1000i urine analyzer, but it was higher in bladder cancer (8.7%, p < 0.001) than in glomerular disease (1.9%) in the data obtained via the Cobas 6500 urine analyzer.

## Discussion

The urine dipstick test is very sensitive for the detection of hematuria (sensitivity 91–100%), but it may not be specific (specificity 65–99%) because it also yields positive results for hemoglobinuria or myoglobinuria^25^. In the present study, the participating subjects showed no clinical evidence of intravascular hemolysis or rhabdomyolysis, and a positive urine dipstick blood test was strongly indicative of hematuria considering that bladder cancer and glomerular disease had been pathologically proven. For both bladder cancer and glomerular disease, the grades of hematuria measured using the dipstick were well correlated with urine RBC counts measured via the automated urine sediment analyzers. In dilute or highly alkaline urine, RBCs may undergo lysis. Even after dilute or alkaline urine specimens were excluded, however, there were urinalyses with a positive dipstick test for blood but negative or relatively few RBCs, suggesting that some RBCs are degraded before urinalysis or are not detected. Such urinalyses were more frequent in cases of glomerular disease than in cases of bladder cancer, and at a trace, 1+, or higher positive dipstick tests for blood, the RBC counts were significantly lower in glomerular disease than in bladder cancer.

In glomerular hematuria, RBCs pass through the glomerular filtration barrier and the convoluted tubules, which may distort the RBC morphology. RBCs may undergo lysis during this passage, or the morphologically altered RBCs can be more vulnerable to hemolysis^26^. If RBCs are degraded, they are not detected by microscopic examination, but the hemoglobin released from the RBCs is still detected by the dipstick test^27^. Besides, automated urine sediment analyzers detect isomorphic RBCs well but may not be good at detecting dysmorphic RBCs^8,28,29^.

The UF-1000i urine analyzer counts RBCs by flow cytometry, whereas the Cobas 6500 urine analyzer identifies RBCs by the analysis of digital images of the urine sediment. Though the two urine sediment analyzers counted fewer RBCs in patients with glomerular disease than in patients with bladder cancer for each positive dipstick blood test, the differences in RBC numbers between patients with bladder cancer and patients with glomerular disease were greater with the Cobas 6500 urine analyzer than with the UF-1000i urine analyzer. This may be in part because the Cobas 6500 urine analyzer tended to count fewer RBCs in the concentrated urine of patients with glomerular disease. The Cobas 6500 urine analyzer may also give some false positive results for RBCs in patients with bladder cancer because 3 or more RBCs/HPF despite a negative result in the dipstick test was more frequent in patients with bladder cancer than in those with glomerular disease.

In urologic malignancies, hematuria is intermittent, and the level of microscopic hematuria does not indicate the seriousness of the disease^30^, and it is important to determine the presence or absence of hematuria. If adults older than 40 years test positive for hematuria, a urologic investigation is required to exclude malignancy. Because the dipstick test detects heme from hemoglobinuria, myoglobinuria, and glomerular hematuria as well as extraglomerular hematuria, there is a high probability of the result being a false positive for the diagnosis of urologic malignancy. Thus, hematuria is defined as ≥ 3 RBCs/HPF and this is the widely accepted definition across urologic societies^3,4^; furthermore, the dipstick hematuria test is not considered sufficient to mandate an evaluation.

In glomerular disease, however, the amount of hematuria may be more important than the presence or absence of hematuria because high-grade hematuria suggests a nephritic glomerular disease in the differential diagnosis and may also indicate a worse renal prognosis.

In a recent study^31^, urine dipstick hematuria was shown to be useful in differentiating proliferative glomerulonephritis from other causes of kidney diseases, with higher positive grades in the dipstick blood test associated with higher positive predictive values.

The association of hematuria with adverse renal outcomes has also been reported in glomerular diseases. IgA nephropathy is one of the representative glomerular diseases presenting with hematuria. The persistence of hematuria in IgA nephropathy was related to a greater probability of developing end-stage kidney disease (ESKD)^14,17^. Similarly, hematuria in chronic kidney disease was associated with a faster decline in kidney function or an increased risk of developing ESKD^15,16^. In diabetic nephropathy, hematuria is usually absent, but its presence was associated with an increased risk of ESKD^18,21^. High-grade hematuria in glomerular disease indicates severe inflammation of glomeruli, and hematuria itself also may increase the injury to the kidney parenchyma by releasing the toxic molecules such as free hemoglobin and heme if RBCs are ruptured while passing through glomeruli and tubules^26^.

In the above studies, hematuria was defined by the RBC numbers. In a few studies where RBC counts were not available, the dipstick test was used to define hematuria. Dipstick hematuria was also shown to be a risk factor for deterioration of glomerular filtration in chronic kidney disease^32,33^ and diabetic nephropathy^34^; however, the grading of hematuria severity was not included in the analysis.

Considering the significance of hematuria in glomerular disease, the severity of hematuria needs to be assessed accurately. However, the current study suggests that automated urine sediment analyzers do not reliably detect RBCs of glomerular origin, and the defining hematuria based on RBC numbers may result in hematuria not being detected or in the severity of hematuria being underestimated in glomerular disease.

Besides the urinalysis method being used, the collection of an adequate urine specimen is a prerequisite for accurate urine sediment examination. To avoid collecting dilute urine, it is recommended that urinalysis be performed using the first-morning specimen. However, randomly voided urine is the most commonly used specimen for urinalysis because it is easiest to obtain, as it was in this study. With the randomly voided urine, 18–22% of the urine specimens had SG < 1.010, which made the assessment of hematuria by RBC counting unreliable due to hypotonic lysis of RBCs.

Even in urine specimens with SG ≥ 1.010, the RBC counts can be affected by the level of urine concentration, with a higher number of RBCs in more concentrated urine. However, the RBC counts are reported without a correction for the degree of urine concentration, and thus do not allow quantitative assessment of hematuria. The same is true for the dipstick blood test.

As compared with urine RBC counting, the urine dipstick blood test may have more advantages for the follow-up of hematuria in cases of glomerular disease, as the results can be positive due to dysmorphic RBCs as well as isomorphic RBCs; further, hematuria can be detected in dilute urine specimens by the presence of heme released from lysed RBCs^27^.

This study has some limitations. First, automated urinary sediment examination was not compared with the manual microscopic examination, which is recommended in the evaluation of urine sediments in patients with glomerular disease. The latter, however, should be performed by experts under standard conditions and is not feasible where a large number of samples need to be processed quickly. Furthermore, RBC counting under the manual microscopic examination is also not accurate and is subject to wide interobserver variability^35^. Second, the dipstick blood test may also give false positives and false negatives. Bacterial peroxidase, for example, can produce false-positive results^36^. However, bacterial peroxidase does not seem to account for the lower numbers of urinary RBCs in glomerular disease because urinary tract infection was not documented in the patients with glomerular disease during the study period. In contrast, ascorbic acid or ascorbic acid-containing soft drinks may cause false-negative results^37^; however, the patients included in this study were not questioned about this before obtaining urinalysis specimens.

In conclusion, our data suggest that urine RBC counting by the UF-1000i urine analyzer or the Cobas 6500 urine analyzer underestimates the severity of hematuria in glomerular disease, possibly because dysmorphic RBCs are susceptible to hemolysis and/or are not properly recognized.

## Supplementary Information

Supplementary Information.
