# Supplementary material for: Automated urine sediment analyzers underestimate the severity of hematuria in glomerular diseases
Source: Sci Rep. 2021 Oct 25;11:20981. doi: 10.1038/s41598-021-00457-6 (PMC8546052; doi:10.1038/s41598-021-00457-6)

# Automated Urine Sediment Analyzers Underestimate the Severity of Hematuria in Glomerular Diseases

Won Seok Yang

Division of Nephrology, Department of Internal Medicine, Asan Medical Center,
University of Ulsan College of Medicine, Seoul, Republic of Korea

## Supplementary Tables & Figures

**Supplementary Table S1.** The distribution of urine RBC counts measured using the UF-1000i urine analyzer at each positive degree of dipstick blood test in the urinalyses (pH < 9.0) of patients with bladder cancer – dilute urine vs. normal urine vs. concentrated urine. SG, specific gravity; RBCs, red blood cells; HPF, high power field.

**A. Urine SG < 1.010**

| Grade | Dipstick blood test | | | | | |
|---|---|---|---|---|---|---|
| (RBCs/HPF) | - | ± | 1+ | 2+ | 3+ | 4+ |
| 7 (>100/HPF) | | | | | | 52 |
| 6 (31-100/HPF) | | | | | 9 | 76 |
| 5 (21-30/HPF) | | | | 5 | 10 | 18 |
| 4 (11-20/HPF) | | | 1 | 16 | 23 | 41 |
| 3 (6-10/HPF) | | | 8 | 12 | 17 | 36 |
| 2 (3-5/HPF) | 2 | 2 | 28 | 18 | 13 | 30 |
| 1 (0-2/HPF) | 505 | 57 | 79 | 47 | 26 | 16 |

**B. Urine SG 1.010 – 1.020**

| Grade | Dipstick blood test | | | | | |
|---|---|---|---|---|---|---|
| (RBCs/HPF) | - | ± | 1+ | 2+ | 3+ | 4+ |
| 7 (>100/HPF) | | | | | 3 | 375 |
| 6 (31-100/HPF) | | | | 6 | 54 | 257 |
| 5 (21-30/HPF) | | | 2 | 28 | 59 | 36 |
| 4 (11-20/HPF) | | | 9 | 84 | 57 | 50 |
| 3 (6-10/HPF) | 1 | 12 | 69 | 73 | 38 | 22 |
| 2 (3-5/HPF) | 14 | 52 | 92 | 52 | 17 | 15 |
| 1 (0-2/HPF) | 1134 | 150 | 148 | 56 | 7 | 6 |

**C. Urine SG > 1.020**

| Grade | Dipstick blood test | | | | | |
|---|---|---|---|---|---|---|
| (RBCs/HPF) | - | ± | 1+ | 2+ | 3+ | 4+ |
| 7 (>100/HPF) | | | | 1 | 2 | 161 |
| 6 (31-100/HPF) | 1 | | | | 18 | 86 |
| 5 (21-30/HPF) | | | | 5 | 18 | 21 |
| 4 (11-20/HPF) | | | 2 | 24 | 10 | 15 |
| 3 (6-10/HPF) | 2 | 2 | 25 | 32 | 13 | 10 |
| 2 (3-5/HPF) | 5 | 13 | 28 | 21 | 5 | 7 |
| 1 (0-2/HPF) | 487 | 44 | 59 | 14 | 5 | 3 |

**Supplementary Figure S1.** The effects of high and low urine specific gravities on the grade of hematuria measured using the UF-1000i urine analyzer at each positive dipstick grade in the urinalyses (pH < 9.0) of patients with bladder cancer. Vertical boxes, error bars, and dots represent the median with interquartile range, minimum and maximum values, and outliers, respectively (***p<0.001). SG, specific gravity.

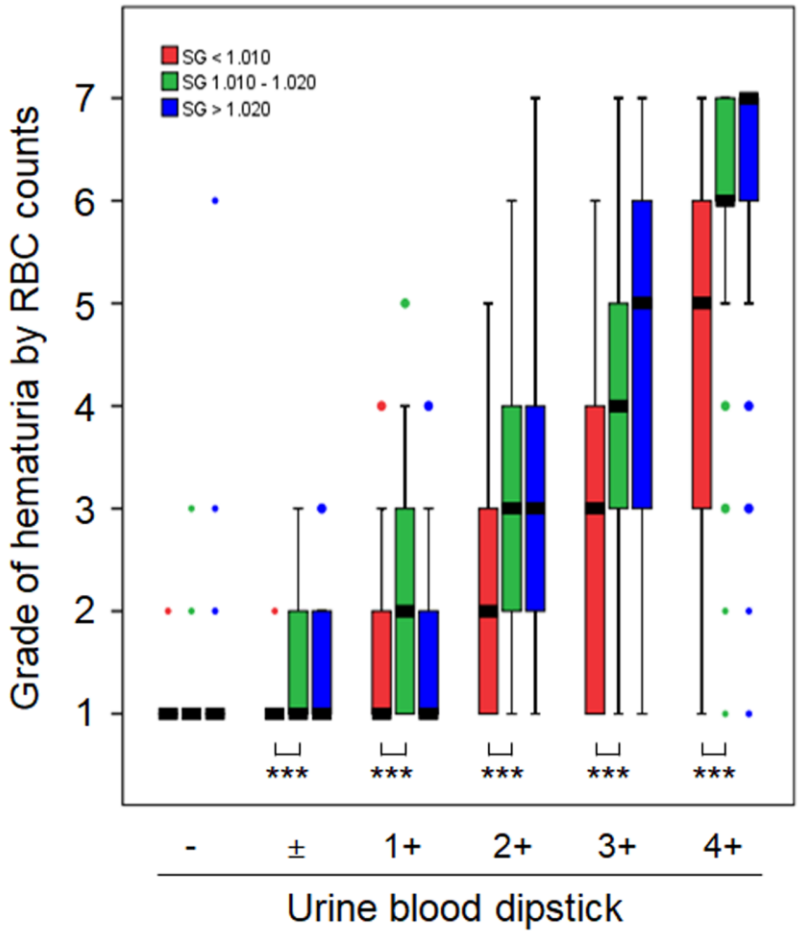

**Supplementary Table S2.** The distribution of urine RBC counts measured using the UF-1000i urine analyzer at each positive degree of dipstick blood test in the urinalyses (pH < 9.0) of patients with glomerular disease – dilute urine vs. normal urine vs. concentrated urine. SG, specific gravity; RBCs, red blood cells; HPF, high power field.

**A. Urine SG < 1.010**

| Grade | Dipstick blood test | | | | | |
|---|---|---|---|---|---|---|
| (RBCs/HPF) | - | ± | 1+ | 2+ | 3+ | 4+ |
| 7 (>100/HPF) | | | | | | 14 |
| 6 (31-100/HPF) | | | | | 1 | 35 |
| 5 (21-30/HPF) | | | | | 3 | 25 |
| 4 (11-20/HPF) | | | | 4 | 10 | 50 |
| 3 (6-10/HPF) | | | 1 | 13 | 39 | 55 |
| 2 (3-5/HPF) | 3 | 3 | 172 | 45 | 35 | 28 |
| 1 (0-2/HPF) | 172 | 48 | 152 | 76 | 27 | 10 |

**B. Urine SG 1.010 – 1.020**

| Grade | Dipstick blood test | | | | | |
|---|---|---|---|---|---|---|
| (RBCs/HPF) | - | ± | 1+ | 2+ | 3+ | 4+ |
| 7 (>100/HPF) | | | | | 1 | 61 |
| 6 (31-100/HPF) | | | | | 11 | 159 |
| 5 (21-30/HPF) | | | | 6 | 18 | 90 |
| 4 (11-20/HPF) | | | 5 | 44 | 91 | 160 |
| 3 (6-10/HPF) | | 2 | 22 | 71 | 88 | 65 |
| 2 (3-5/HPF) | 8 | 14 | 54 | 91 | 76 | 35 |
| 1 (0-2/HPF) | 556 | 142 | 255 | 113 | 39 | 10 |

**C. Urine SG > 1.020**

| Grade | Dipstick blood test | | | | | |
|---|---|---|---|---|---|---|
| (RBCs/HPF) | - | ± | 1+ | 2+ | 3+ | 4+ |
| 7 (>100/HPF) | | | | | | 20 |
| 6 (31-100/HPF) | | | | 1 | 5 | 61 |
| 5 (21-30/HPF) | | | | 2 | 10 | 17 |
| 4 (11-20/HPF) | | | 4 | 17 | 18 | 41 |
| 3 (6-10/HPF) | | 1 | 9 | 16 | 18 | 33 |
| 2 (3-5/HPF) | 6 | 3 | 20 | 26 | 15 | 15 |
| 1 (0-2/HPF) | 205 | 46 | 80 | 35 | 7 | 6 |

**Supplementary Figure S2.** The effects of high and low urine specific gravities on the grade of hematuria measured using the UF-1000i urine analyzer at each positive dipstick grade in the urinalyses (pH < 9.0) of patients with glomerular disease. Vertical boxes, error bars, and dots represent the median with interquartile range, minimum and maximum values, and outliers, respectively (*p<0.05, ***p<0.001). SG, specific gravity.

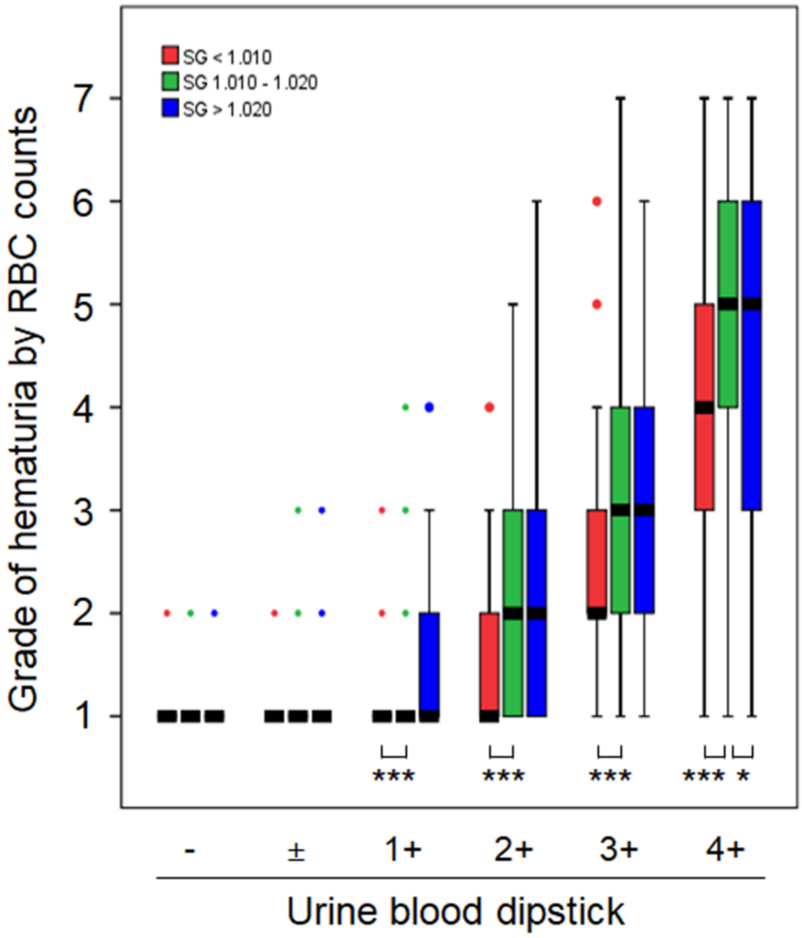

**Supplementary Table S3.** The distribution of urine RBC counts measured using the Cobas 6500 urine analyzer at each positive degree of dipstick blood test in the urinalyses (pH < 9.0) of patients with bladder cancer – dilute urine vs. normal urine vs. concentrated urine. SG, specific gravity; RBCs, red blood cells; HPF, high power field.

A. Urine SG < 1.010

| Grade | Dipstick blood test | | | | | |
|---|---|---|---|---|---|---|
| (RBCs/HPF) | - | ± | 1+ | 2+ | 3+ | 4+ |
| 7 (>100/HPF) | | | | | | 100 |
| 6 (31-100/HPF) | | | | 3 | 19 | 58 |
| 5 (21-30/HPF) | | | 3 | 4 | 8 | 25 |
| 4 (11-20/HPF) | | | 7 | 12 | 12 | 32 |
| 3 (6-10/HPF) | 4 | 13 | 5 | 15 | 19 | 23 |
| 2 (3-5/HPF) | 24 | 14 | 1 | 19 | 10 | 26 |
| 1 (0-2/HPF) | 723 | 47 | 1 | 36 | 19 | 27 |

B. Urine SG 1.010 – 1.020

| Grade | Dipstick blood test | | | | | |
|---|---|---|---|---|---|---|
| (RBCs/HPF) | - | ± | 1+ | 2+ | 3+ | 4+ |
| 7 (>100/HPF) | | | | | 11 | 651 |
| 6 (31-100/HPF) | 1 | 1 | | 22 | 166 | 298 |
| 5 (21-30/HPF) | | 1 | 8 | 42 | 71 | 43 |
| 4 (11-20/HPF) | 2 | 21 | 35 | 91 | 54 | 44 |
| 3 (6-10/HPF) | 16 | 100 | 20 | 49 | 32 | 11 |
| 2 (3-5/HPF) | 145 | 86 | 7 | 47 | 13 | 5 |
| 1 (0-2/HPF) | 1777 | 171 | 9 | 47 | 9 | 3 |

C. Urine SG > 1.020

| Grade | Dipstick blood test | | | | | |
|---|---|---|---|---|---|---|
| (RBCs/HPF) | - | ± | 1+ | 2+ | 3+ | 4+ |
| 7 (>100/HPF) | | 1 | | | 6 | 326 |
| 6 (31-100/HPF) | | | | 4 | 67 | 124 |
| 5 (21-30/HPF) | | | 1 | 17 | 19 | 18 |
| 4 (11-20/HPF) | | 8 | 5 | 28 | 27 | 13 |
| 3 (6-10/HPF) | 7 | 44 | 20 | 27 | 12 | 10 |
| 2 (3-5/HPF) | 72 | 41 | 2 | 19 | 8 | 6 |
| 1 (0-2/HPF) | 769 | 78 | 5 | 8 | 9 | 4 |

**Supplementary Figure S3.** The effects of high and low urine specific gravities on the grade of hematuria measured using the Cobas 6500 urine analyzer at each positive dipstick grade in the urinalyses (pH < 9.0) of patients with bladder cancer. Vertical boxes, error bars, and dots represent the median with interquartile range, minimum and maximum values, and outliers, respectively (*p<0.05, **p<0.01, ***p<0.001). SG, specific gravity.

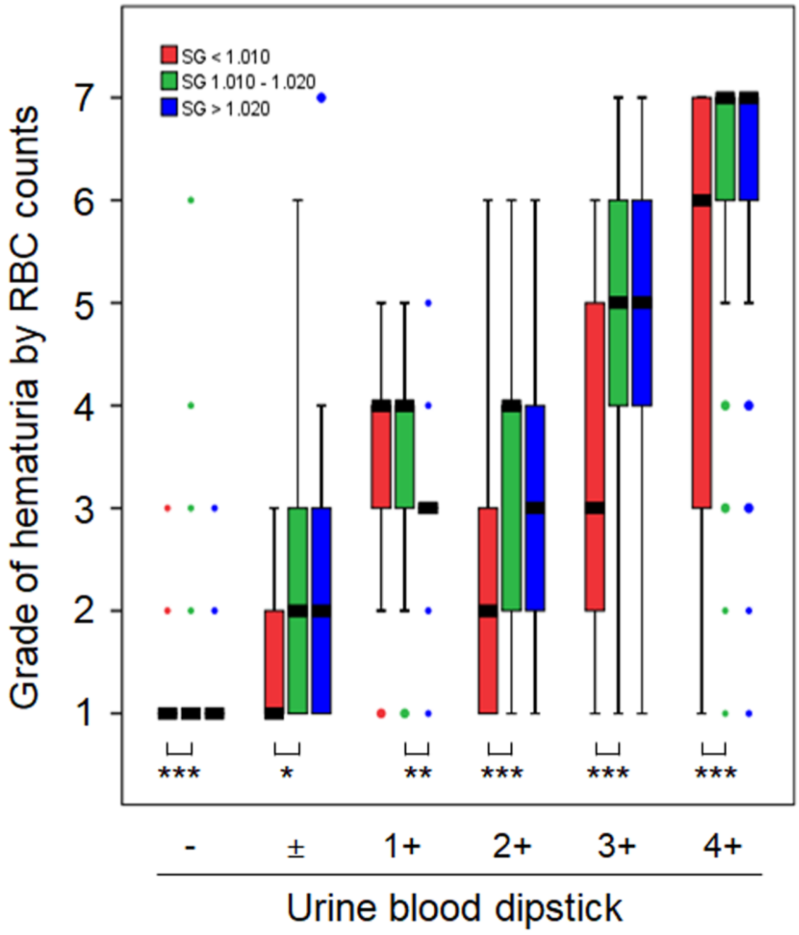

**Supplementary Table S4.** The distribution of urine RBC counts measured using the Cobas 6500 urine analyzer at each positive degree of dipstick blood test in the urinalyses (pH < 9.0) of patients with glomerular disease – dilute urine vs. normal urine vs. concentrated urine. SG, specific gravity; RBCs, red blood cells; HPF, high power field.

A. Urine SG < 1.010

| Grade | Dipstick blood test | | | | | |
|---|---|---|---|---|---|---|
| (RBCs/HPF) | - | ± | 1+ | 2+ | 3+ | 4+ |
| 7 (>100/HPF) | | | | | | 21 |
| 6 (31-100/HPF) | | | | | 1 | 51 |
| 5 (21-30/HPF) | | | | | 3 | 31 |
| 4 (11-20/HPF) | | | | 2 | 12 | 35 |
| 3 (6-10/HPF) | | | | 10 | 35 | 28 |
| 2 (3-5/HPF) | 1 | 17 | | 33 | 27 | 18 |
| 1 (0-2/HPF) | 196 | 71 | 11 | 46 | 37 | 9 |

B. Urine SG 1.010 – 1.020

| Grade | Dipstick blood test | | | | | |
|---|---|---|---|---|---|---|
| (RBCs/HPF) | - | ± | 1+ | 2+ | 3+ | 4+ |
| 7 (>100/HPF) | | | | | | 134 |
| 6 (31-100/HPF) | | | | | 17 | 226 |
| 5 (21-30/HPF) | | | | 2 | 26 | 93 |
| 4 (11-20/HPF) | | | 2 | 22 | 49 | 129 |
| 3 (6-10/HPF) | | 12 | 2 | 39 | 67 | 64 |
| 2 (3-5/HPF) | 14 | 55 | 13 | 60 | 59 | 48 |
| 1 (0-2/HPF) | 714 | 215 | 23 | 106 | 64 | 11 |

C. Urine SG > 1.020

| Grade | Dipstick blood test | | | | | |
|---|---|---|---|---|---|---|
| (RBCs/HPF) | - | ± | 1+ | 2+ | 3+ | 4+ |
| 7 (>100/HPF) | | | | | | 12 |
| 6 (31-100/HPF) | | | | | 5 | 77 |
| 5 (21-30/HPF) | | | | 1 | 3 | 37 |
| 4 (11-20/HPF) | | | | 4 | 7 | 44 |
| 3 (6-10/HPF) | | 1 | | 9 | 21 | 24 |
| 2 (3-5/HPF) | 6 | 13 | | 16 | 19 | 33 |
| 1 (0-2/HPF) | 292 | 78 | 6 | 52 | 40 | 15 |

**Supplementary Figure S4.** The effects of high and low urine specific gravities on the grade of hematuria measured using the Cobas 6500 urine analyzer at each positive dipstick grade in the urinalyses (pH < 9.0) of patients with glomerular disease. Vertical boxes, error bars, and dots represent the median with interquartile range, minimum and maximum values, and outliers, respectively (*p<0.05, **p<0.01, ***p<0.001). SG, specific gravity.

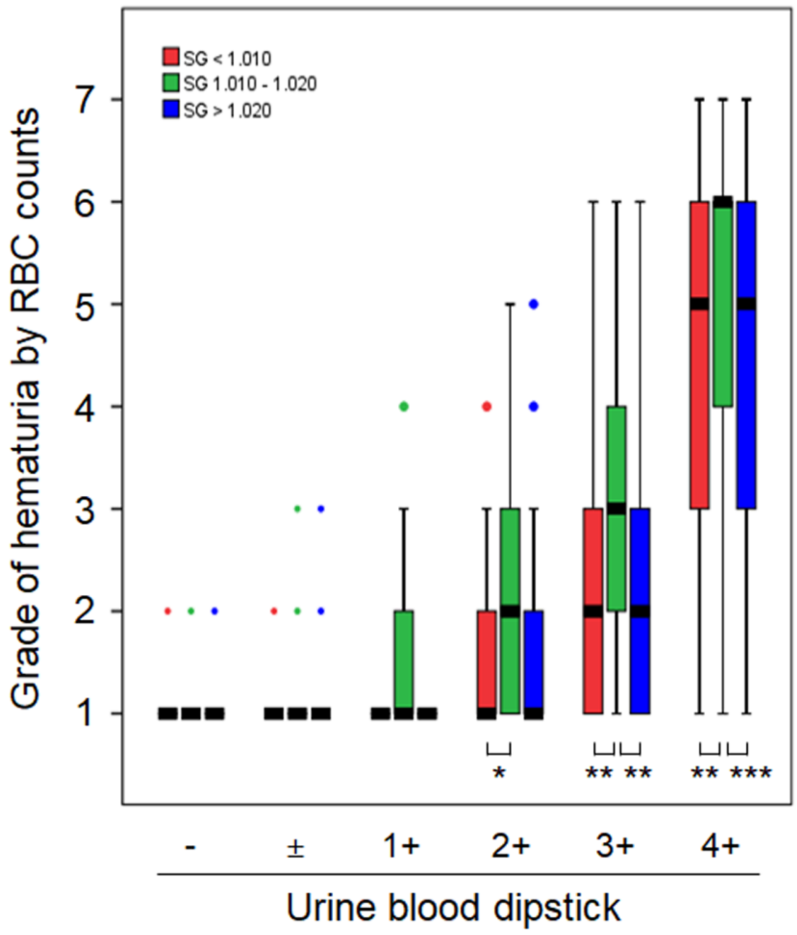

Supplement: Supplementary file 1 — Supplementary Information. [file 41598_2021_457_MOESM1_ESM.pdf]
